# Effects of Body Fat on the Associations of High-Molecular-Weight Adiponectin, Leptin and Soluble Leptin Receptor with Metabolic Syndrome in Chinese

**DOI:** 10.1371/journal.pone.0016818

**Published:** 2011-02-15

**Authors:** Danxia Yu, Zhijie Yu, Qi Sun, Liang Sun, Huaixing Li, Jun Song, Ming Mi, Hongyu Wu, Ling Lu, Chen Liu, Geng Zhang, Frank B. Hu, Xu Lin

**Affiliations:** 1 Key Laboratory of Nutrition and Metabolism, Institute for Nutritional Sciences, Shanghai Institutes for Biological Sciences, Chinese Academy of Sciences, Shanghai, China; 2 Department of Nutrition, Harvard School of Public Health, Boston, Massachusetts, United States of America; 3 Shanghai Municipal Center for Disease Control and Prevention, Shanghai, China; 4 Department of Epidemiology, Harvard School of Public Health, Boston, Massachusetts, United States of America; National Institutes of Health - National Institute of Child Health and Human Development, United States of America

## Abstract

**Background:**

Little is known regarding the associations between high-molecular-weight (HMW-) adiponectin, leptin and soluble leptin receptor (sOB-R) and metabolic syndrome (MetS) in Chinese. Also few studies elucidate the effects of inflammation and body fat mass on the relations.

**Methods:**

Plasma HMW-adiponectin, leptin and sOB-R were measured among 1055 Chinese men and women (35∼54 yrs). Whole body and trunk fat mass were determined by Dual-energy X-ray absorptiometry. MetS was defined by the updated NCEP/ATPIII criterion for Asian-Americans.

**Results:**

HMW-adiponectin was inversely associated with MetS in multivariate model including fat mass index (FMI), inflammatory markers, leptin and sOB-R (OR in the highest quartile  = 0.30, 95%CI 0.18∼0.50, *P*<.0001). Plasma sOB-R was also inversely associated with MetS independent of body fatness and inflammatory markers, whereas the association was somewhat attenuated after adjusting HMW-adiponectin (OR for the highest quartile  = 0.78, 95%CI 0.47∼1.32, *P* = 0.15). In contrast, leptin was associated with increased odds of MetS independent of inflammatory markers, HMW-adiponectin, and sOB-R (OR for the highest quartile  = 2.64, 95%CI 1.35∼5.18, *P* = 0.006), although further adjustment for FMI abolished this association.

**Conclusions:**

HMW-adiponectin exhibited strong inverse associations with MetS independent of body composition, inflammation, leptin and sOB-R; while the associations of leptin and sOB-R were largely explained by fat mass or HMW-adiponectin, respectively.

## Introduction

Adipose tissue is an endocrine organ playing a pivotal role in the pathogenesis of metabolic diseases. Several adipose-derived adipokines have been demonstrated as mediators linking obesity to insulin resistance, dyslipidemia and inflammation [1]. Adiponectin, one of the most abundant adipokines in circulation, exhibits insulin-sensitizing, fat-burning and anti-inflammatory properties [2], [3]. Hypoadiponectinmia frequently appeared in Individuals with obesity, metabolic syndrome (MetS) and type 2 diabetes [4], [5]. However, there are at least 3 isomers of adiponectin, *i.e.*, trimer, hexamer and high-molecular-weight (HMW-) multimer [2]. Accumulating evidence suggests that HMW-adiponectin is the most physiologically active form related to glucose tolerance [6], insulin sensitivity [7], central fat distribution and multiple metabolic disorders [8]. Previous studies also indicated that HMW-adiponectin could be a useful marker to evaluate risk of MetS or type 2 diabetes in elderly Japanese [9], Japanese-Americans [10] or Caucasian women [11].

Another major and well studied adipokine in blood stream is leptin which could suppress food intake and stimulate energy expenditure [12]. However, rather than leptin deficient, obese persons often have hyperleptinemia specified as “selective leptin resistance” associated with MetS, type 2 diabetes and cardiovascular disease (CVD) [13], [14]. Circulating leptin is either in a free form which could trigger downstream signaling or in a binding form with its soluble receptor (sOB-R). Recently, a large prospective cohort reported a strong inverse association between sOB-R and diabetes, independent of BMI, leptin and adiponectin [15]. However, data regarding the associations of leptin and sOB-R and MetS, the important risk factor of diabetes and CVD, is rather limited and inconsistent [14]–[19].

Adipokines dysregulation has been considered as one of the major mechanisms mediating adverse effects of excess body fat on metabolic abnormalities. However, most studies so far have used BMI to evaluate adiposity and it remains unclear how fat mass or fat distribution *per se* influences the associations between the adipokines and metabolic disorders. Moreover, body composition may also vary according to ethnical background. Compared to Caucasians, Asians are more likely to have abdominal obesity under “normal BMI” [20] and prone to type 2 diabetes at lower BMI [21]. Meanwhile, accompanying rapid diet and lifestyle transition, epidemic trend of metabolic diseases has become a major public health problem in China [22], which is estimated to have 92.4 million adults with diabetes and another 148.2 million people with prediabetes [23]. Obviously, understanding the roles of these adipokines and also their modifying factors could be critical for metabolic disease control and prevention in countries like China.

Therefore, our primary aim was to examine the associations of plasma HMW-adiponectin, leptin and sOB-R with risk of MetS and its components. Meanwhile, we also evaluated how these associations were modified by BMI, inflammatory markers, body fat mass or trunk fat percentage and other established risk factors in 1055 middle-aged Chinese men and women.

## Methods

### Study design and subjects

The study population was non-institutionalized residents from The Gut Microbiota and Obesity Study, a case-control study of normal weight (18≤ BMI <24.0 kg/m^2^) and overweight/obesity (BMI ≥24.0 kg/m^2^) participants [22] aged from 35 to 54 years living in Shanghai for at least 10 years. Detailed study design and inclusion/exclusion criteria were described elsewhere [24]. Four men with missing values for adipokines were excluded. The final analytical sample comprised 557 overweight/obese and 498 normal-weight subjects. The protocol was approved by the institutional review board of the Institute for nutritional sciences, Shanghai Institutes for Biological Sciences, Chinese Academy of Sciences. Written informed consents were obtained from all participants.

Information of demographics, health status, diet and lifestyles was collected using a standardized questionnaire during home interview. Dietary intake was assessed with a modified food frequency questionnaire used in the National Survey on the Status of Nutrition and Health of the Chinese People in 2002 [25]. Smoking and drinking were defined as “yes” or “no”. Family history of chronic diseases was defined as one of the parents or siblings having CVD, stroke or type 2 diabetes. Educational attainment was categorized according to self-reported school years. Levels of physical activity were calculated as a sum of metabolic equivalent (MET)-minute/week score [26] and then classified as low, moderate and high. Sleeping was assessed by average daily sleep time.

All participants had a physical examination after overnight fasting. Body weight, height, waist circumference, blood pressure were measured according to a standardized protocol described elsewhere [27]. A dual-energy X-ray absorptiometry scan (DEXA, QDR-4500, Hologic, Waltham, MA, USA) was conducted in 956 (90.6%) individuals and no significant difference in characteristics was found between those with and without DEXA. Values for total fat mass, trunk fat mass and trunk mass (kg) were obtained by using software built into the scanner (version 11.2.1) and daily quality control was performed using phantom #12447 the same as previous study [28].

### Laboratory measurements

The blood processing procedure and assays for fasting plasma glucose, insulin, triglycerides, HDL cholesterol (HDL-C), high-sensitive C-reactive protein (hsCRP) and IL-6 were described in a previous study [29].

HMW-adiponectin was assessed using an ELISA kit (Millipore, St. Charles, MO, USA). Leptin and sOB-R were determined also by ELISA (R&D Systems Inc., Minneapolis, MN, USA). The average intra-assay and inter-assay coefficients of variation (CVs) were <10%.

### Ascertainment of metabolic syndrome

Metabolic syndrome was defined according to the updated National Cholesterol Education Program Adult Treatment Panel III criteria for Asian-Americans [30], which includes at least three of the following components: 1) waist circumferences ≥90 cm in men or ≥80 cm in women; 2) triglycerides ≥1.7 mmol/L; 3) HDL-C <1.03 mmol/L in men or <1.30 mmol/L in women; 4) blood pressure ≥130/85 mmHg, or current use of anti-hypertensive medications; 5) fasting plasma glucose ≥5.6 mmol/L.

### Statistical analysis

Fat mass index (FMI) was calculated based upon DEXA data as total fat mass (kg)/height (m)^2^ [28]. Trunk fat percentage was the ratio of trunk fat mass (kg) to total trunk mass (kg). Homeostasis model assessment of insulin resistance (HOMA-IR) was computed using updated homeostasis model assessment methods (http://www.dtu.ox.ac.uk/).

All continuous variables were log-transformed to improve normality. General linear model was applied for the comparisons between non-MetS and MetS (Table S1). Spearman partial correlation coefficients were estimated after adjustment for age, sex and other covariates (Table 1). Because of gender differences in the distributions of adipokines, sex-specific quartile cut-points were used. Multivariate logistic regression models were used to estimate the odds ratios, adjusting for age (continuous), sex, newly diagnosed diabetes (fasting plasma glucose ≥7.0 mmol/L or 2-h post load plasma glucose ≥11.1 mmol/L), smoke (past/current or not), current alcohol use (yes or not), family history of chronic diseases (yes or not), education (0∼9, 10∼12 or >12 years), physical activity (low, moderate or high), sleep (<7, 7∼9 or ≥9 h/d), total energy intake (kcal/d), BMI or FMI or trunk fat percentage, hsCRP, IL-6 and adipokines (log-transformed, continuous). All statistical analyses were performed using Stata 9.2 (Stata, College Station, TX, USA). Two-sided *P*<0.05 was considered statistically significant.

**Table 1:** Spearman partial correlation coefficients with metabolic parameters 1.

	HMW-adiponectin(µg/mL)	Leptin(ng/mL)	sOB-R(ng/mL)
BMI (kg/m^2^) 2, 3	−0.31	0.66	−0.31
FMI (kg/m^2^) 2	−0.27	0.75	−0.31
Trunk fat percentage (%)	−0.18	−0.04^ns^	−0.05^ns^
Waist circumference (cm)	−0.19	−0.02^ns^	−0.13
Systolic blood pressure (mm Hg)	−0.06^ns^	−0.03^ns^	0.09
Diastolic blood pressure (mm Hg)	−0.02^ns^	−0.006^ns^	0.06^ns^
Fasting glucose (mmol/L)	−0.03^ns^	0.04^ns^	0.04^ns^
Insulin (µU)	−0.22	0.13	−0.16
HOMA-IR	−0.22	0.13	−0.15
Triglycerides (mmol/L)	−0.28	0.09	−0.09
HDL cholesterol (mmol/L)	0.29	0.01^ns^	0.17
hsCRP (mg/L)	−0.05^ns^	0.03^ns^	−0.009^ns^
IL-6 (pg/mL)	−0.08	0.008^ns^	−0.11
HMW-Adiponectin (µg/mL)	1	-	-
Leptin (ng/mL)	0.02^ns^	1	-
sOB-R (ng/mL)	0.17	−0.002^ns^	1

1 Analyses were conducted in 956 participants. All coefficients were significant unless indicated “ns”. Adjusted for age, sex, diabetes, smoke, alcohol, family history of chronic diseases, education, physical activity, sleep, total energy intake and fat mass index (FMI).

2 Adjusted all abovementioned variables except FMI.

3 Analyses were conducted in 1055 participants.

## Results

Individuals with MetS were older, and had higher BMI, waist circumference, blood pressure, fasting glucose, insulin, HOMA-IR, triglycerides, inflammatory markers (hsCRP and IL-6), fat mass index and trunk fat percentage, but lower HDL-C. Meanwhile, they also exhibited lower concentrations of HMW-adiponectin and sOB-R, but greater leptin compared with those without MetS (Table S1).

HMW-adiponectin was inversely associated with BMI (Table 1, r = −0.31) and FMI (r = −0.27). Controlling for FMI, HMW-adiponectin was still associated negatively with trunk fat percentage (r = −0.18), waist circumference (r = −0.19), insulin (r = −0.22), HOMA-IR (r = −0.22), triglycerides (r = −0.28) and IL-6 (r = −0.08), while positively with HDL-C (r = 0.29) and sOB-R (r = 0.17). In comparison, sOB-R showed somewhat weaker associations: r = −0.13 with waist circumference, r = −0.09 with triglycerides, r = 0.17 with HDL-C and no relation to trunk fat percentage (r = −0.05).

In contrast, leptin was strongly correlated with BMI (r = 0.66) and FMI (r = 0.75). After adjustment for FMI, only the correlations with insulin (r = 0.13), HOMA-IR (r = 0.13), triglycerides (r = 0.09), remained statistically significant.

### Risk of metabolic syndrome

**Figure 1:**
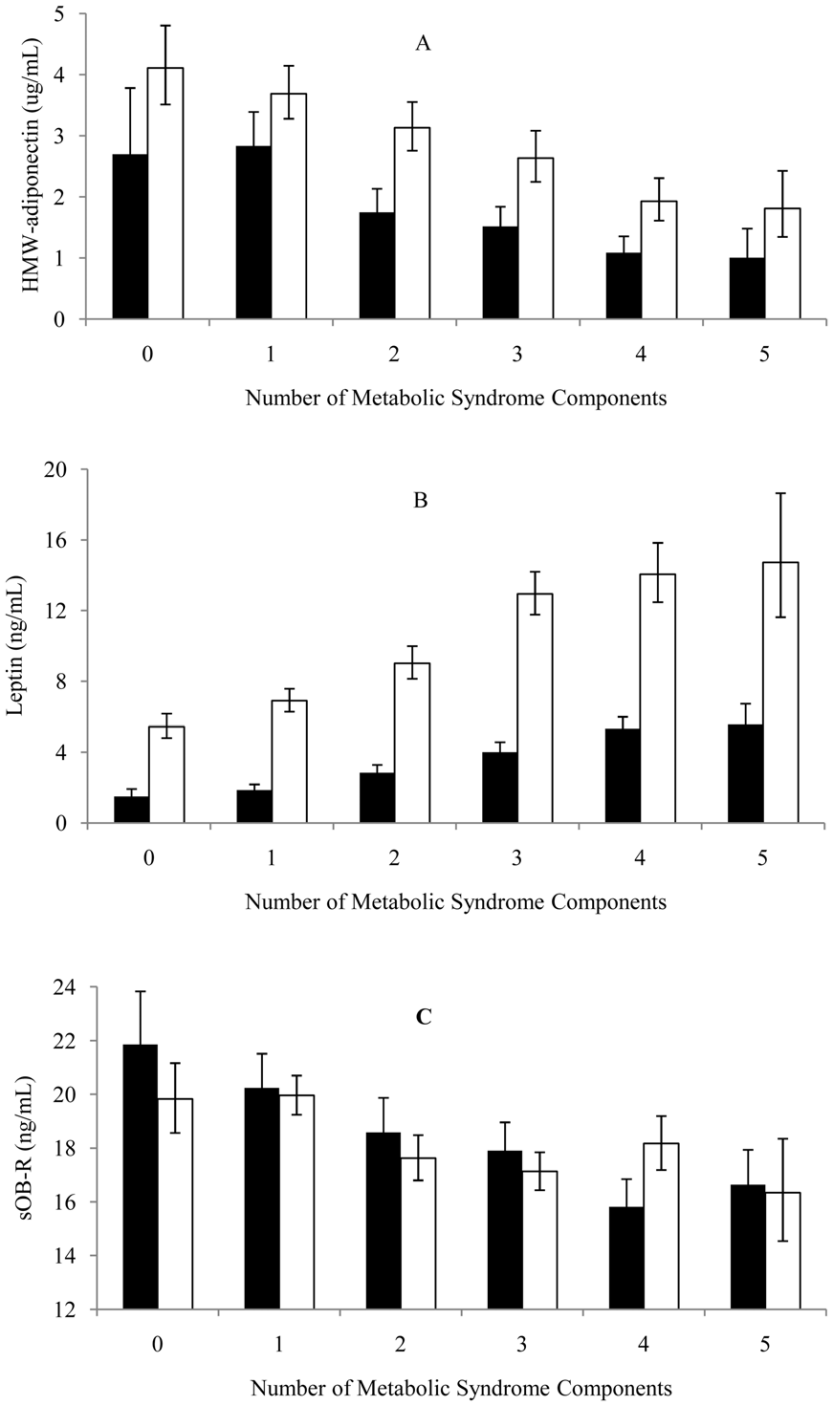
Geometric means (95%CI) of concentrations of HMW-adiponectin (A), leptin (B) and soluble leptin receptor (sOB-R, C) in individuals with 0 to 5 components of metabolic syndrome. Black bars  =  Men; white bars  =  Women. All *P* values were <.0001.

HMW-adiponectin and sOB-R decreased (Figure 1, A and C) while leptin increased (Figure 1B) with an increased number of MetS components in both men and women (all *P*<.0001).

In multivariate logistic regression analyses, both HMW-adiponectin and sOB-R were negatively, whereas leptin was positively, associated with the risk of MetS independent of BMI and inflammatory markers (Table 2, Model 1 and 2). The odds ratios (ORs) comparing the highest with the lowest quartile were 0.34 (95%CI 0.20∼0.58, *P*_trend_<.0001) for HMW-adiponectin, 0.68 (95%CI 0.41∼1.13, *P*_trend_ = 0.05) for sOB-R and 2.60 (95%CI 1.34∼5.05, *P*_trend_ = .005) for leptin. Further adjustments of leptin and sOB-R showed little impact on the association between HMW-adiponectin and MetS. Similarly, adjustment for sOB-R and HMW-adiponectin did not affect the association between leptin and MetS. However, the significant association between sOB-R and MetS disappeared after HMW-adiponectin was included in the Model 3 (*P*_trend_ = 0.15). Replacing BMI with FMI (Model 4) did not substantially change the significant associations for both HMW-adiponectin and sOB-R (ORs in the highest quartile were 0.30, 95%CI 0.18∼0.50, *P*_trend_<.0001 and 0.61, 95%CI 0.36∼1.02, *P*_trend_ = 0.02, accordingly); but abolished the significance between leptin and the risk of MetS (*P*_trend_ = 0.23).

**Table 2:** Odds ratio (95%CI) of metabolic syndrome according to sex-specific quartile of HMW-adiponectin, leptin and sOB-R.

Adipokines	Q1	Q2	Q3	Q4	*P* for trend
**HMW-adiponectin** 1					
Model 1 2	1	0.66 (0.41, 1.04)	0.46 (0.29, 0.74)	0.34 (0.20, 0.56)	<.0001
Model 2 2	1	0.65 (0.41, 1.04)	0.46 (0.29, 0.74)	0.34 (0.20, 0.58)	<.0001
Model 3 2	1	0.64 (0.40, 1.02)	0.46 (0.29, 0.74)	0.35 (0.21, 0.59)	<.0001
Model 4 3	1	0.60 (0.37, 0.96)	0.49 (0.30, 0.79)	0.30 (0.18, 0.50)	<.0001
**Leptin** 1					
Model 1 2	1	1.89 (1.07, 3.34)	2.76 (1.51, 5.01)	3.02 (1.57, 5.78)	0.0009
Model 2 2	1	1.84 (1.03, 3.28)	2.60 (1.41, 4.78)	2.60 (1.34, 5.05)	0.005
Model 3 2	1	1.86 (1.03, 3.36)	2.57 (1.39, 4.75)	2.64 (1.35, 5.18)	0.006
Model 4 3	1	1.44 (0.80, 2.59)	1.70 (0.92, 3.17)	1.59 (0.78, 3.24)	0.23
**sOB-R** 1					
Model 1 2	1	0.92 (0.58, 1.44)	0.61 (0.38, 0.98)	0.59 (0.36, 0.97)	0.01
Model 2 2	1	0.99 (0.62, 1.57)	0.64 (0.40, 1.04)	0.68 (0.41, 1.13)	0.05
Model 3 2	1	1.07 (0.67, 1.71)	0.70 (0.43, 1.13)	0.78 (0.47, 1.32)	0.15
Model 4 3	1	0.82 (0.52, 1.32)	0.56 (0.34, 0.91)	0.61 (0.36, 1.02)	0.02

1 Model 1: adjusted for age, sex, diabetes, smoke, alcohol, family history of chronic diseases, education, physical activity, sleep, total energy intake and log-transformed BMI.

Model 2: model 1+ inflammatory factors (hsCRP and IL-6).

Model 3: model 2+ other adipokines (leptin and sOB-R or HMW-adiponectin).

Model 4: adjusted for log-transformed FMI instead of BMI in Model 1.

2 Quartiles of HMW-adiponectin (µg/mL) were <1.08, 1.08–1.84, 1.84–3.16, >3.16 for men and <1.86, 1.86–3.15, 3.15–5.33, >5.33 for women. Quartiles of leptin (ng/mL) were <1.95, 1.95–3.22, 3.22–5.58, >5.58 for men and <5.92, 5.92–9.64, 9.64–15.21, >15.21 for women. Quartiles of sOB-R (ng/mL) were <15.16, 15.16–18.19, 18.19–22.15, >22.15 for men and <15.54, 15.54–18.45, 18.45–21.82, >21.82 for women.

3 Data were available for 956 participants.

With respect to individual components of MetS (Table 3), HMW-adiponectin was strongly associated with a decreased risk of hypertriglyceridemia (OR in the highest quartile  = 0.26, 95%CI 0.16∼0.42, *P*_trend_<.0001) and low HDL-C (OR in the highest quartile  = 0.22, 95%CI 0.14∼0.35, *P*_trend_<.0001), while marginally associated with central obesity (OR in the highest quartile  = 0.49, 95%CI 0.25∼0.97, *P*_trend_ = 0.06) in the FMI-adjusted model. Furthermore, trunk fat percentage adjustment did not affect these associations substantially (data not shown). Meanwhile, sOB-R was negatively associated with abdominal fat (OR in the highest quartile  = 0.36, 95%CI 0.18∼0.73, *P*_trend_ = 0.002), high triglycerides (OR in the highest quartile  = 0.64, 95%CI 0.40∼1.01, *P*_trend_ = 0.02) and low HDL-C (OR in the highest quartile  = 0.47, 95%CI 0.31∼0.73, *P*_trend_<.0001). In contrast, leptin was only positively associated with hypertriglyceridemia after adjustment for FMI (OR in the highest quartile  = 2.90, 95%CI 1.46∼5.77, *P*_trend_ = 0.006).

**Table 3:** Odds ratio (95%CI) of individual metabolic syndrome component according to sex-specific quartile of HMW-adiponectin, leptin and sOB-R 1.

Adipokines	Q1	Q2	Q3	Q4	*P* for Trend 2
**HMW-adiponectin**					
Central obesity	1	0.65 (0.35, 1.21)	0.68 (0.35, 1.31)	0.49 (0.25, 0.97)	0.06
Hyperglycemia	1	0.93 (0.61, 1.41)	0.80 (0.53, 1.22)	0.88 (0.58, 1.35)	0.45
Elevated blood pressure	1	0.69 (0.46, 1.05)	0.99 (0.65, 1.50)	0.88 (0.57, 1.35)	0.96
Hypertriglyceridemia	1	0.68 (0.45, 1.03)	0.39 (0.25, 0.60)	0.26 (0.16, 0.42)	<.0001
Reduced HDL-C	1	0.78 (0.53, 1.13)	0.54 (0.36, 0.79)	0.22 (0.14, 0.35)	<.0001
**Leptin**					
Central obesity	1	0.60 (0.30, 1.23)	0.84 (0.40, 1.76)	0.50 (0.20, 1.30)	0.44
Hyperglycemia	1	1.48 (0.97, 2.25)	1.74 (1.06, 2.86)	1.50 (0.83, 2.73)	0.15
Elevated blood pressure	1	0.97 (0.61, 1.53)	0.76 (0.45, 1.27)	0.83 (0.45, 1.53)	0.43
Hypertriglyceridemia	1	2.12 (1.21, 3.70)	2.76 (1.51, 5.05)	2.90 (1.46, 5.77)	0.006
Reduced HDL-C	1	0.96 (0.61, 1.50)	0.88 (0.53, 1.46)	0.90 (0.50, 1.61)	0.69
**sOB-R**					
Central obesity	1	0.56 (0.29, 1.09)	0.34 (0.17, 0.66)	0.36 (0.18, 0.73)	0.002
Hyperglycemia	1	1.24 (0.82, 1.87)	0.88 (0.58, 1.33)	1.32 (0.86, 2.03)	0.47
Elevated blood pressure	1	0.75 (0.50, 1.14)	1.02 (0.67, 1.55)	1.05 (0.68, 1.62)	0.54
Hypertriglyceridemia	1	0.87 (0.57, 1.33)	0.65 (0.42, 1.01)	0.64 (0.40, 1.01)	0.02
Reduced HDL-C	1	0.88 (0.60, 1.28)	0.57 (0.38, 0.84)	0.47 (0.31, 0.73)	<.0001

1 Number of cases: central obesity (496), hyperglycemia (616), elevated blood pressure (395), hypertriglyceridemia (305), reduced HDL-C (331).

2 Adjusted for the same variables as Model 4 in Table 2, including FMI. Analyses were conducted in 956 participants.

### Stratified analyses

Considering the obesity case-control design of this study and to explore how obesity, total fat mass and trunk fat percentage influenced the observed relationships, we conducted BMI-stratified analyses. In both normal-weight and overweight group, HMW-adiponectin showed strong inverse associations with modified MetS, regardless whether BMI, FMI or trunk fat percentage was adjusted (Table S2, Model 1 to 3). However, leptin was not significantly associated with modified MetS under control of total or abdominal adiposity. A negative association between sOB-R and modified MetS was observed in normal weight individuals only (Model 1), and the significant association disappeared following adjustments of FMI or trunk fat percentage (Model 2 and 3). The results remained essentially the same and no significant interaction was found in consequent subgroup analyses according to gender, FMI, hsCRP and HOMA-IR (Table S3).

## Discussion

Among 1055 middle-aged Chinese men and women, we observed that reduced plasma HMW-adiponectin and sOB-R, and elevated leptin level were significantly associated with an increased risk of MetS and some of its components independent of multiple confounders including BMI and inflammatory markers. However, body fat mass or adipokines showed different modifying effects on these associations. Unlike the association between HMW-adiponectin and MetS which was unaffected by adjusting for fat depots or other adipokines, the positive associations between leptin and MetS were mainly explained by total fat mass, while the associations between sOB-R and MetS were largely influenced by HMW-adiponectin.

### HMW-adiponectin and metabolic syndrome

Our study indicated that low plasma HMW-adiponectin was a strong and independent risk factor related to MetS in Chinese when diet, lifestyles, adiposity, inflammatory factors leptin and sOB-R were extensively controlled. The inverse associations of adiponectin with metabolic diseases and type 2 diabetes have been well established [4], [5]. However, most studies only measured total adiponectin rather than its high-molecular-weight multimer (12–18mer), which may be more biologically active than trimer and hexamer [6], [7], [8]. Evidence regarding the associations between HMW-adiponectin and MetS was sparse and most of them were limited by small sample size and residual confounding. In one relatively large-scale study, Tabar et al. [9] reported inverse associations between HMW-adiponectin and MetS and its components, except high blood pressure in middle-aged to elderly Japanese. Likewise, our study also supported such associations in Chinese population. On the other hand, we found that low HMW-adiponectin concentration was only significantly associated with high triglyceride and low HDL-C, but not with elevated glucose and central obesity. This minor discrepancy could be due to the different definitions for MetS between two studies. It is also plausible that more confounders were controlled in our analyses.

One interesting observation in current study is that associations between HMW-adiponectin and MetS were independent of adiposity measured by BMI, FMI, or trunk fat percentage. In the Nurses' Health Study, Heidemann et al. reported that higher HMW-adiponectin was associated with lower insulin and diabetes risk independent of BMI or waist circumference [11]. While our data demonstrated that significant negative correlations between HMW-adiponectin and insulin or HOMA-IR (both r = −0.22) were independent of FMI. Moreover, adjustment for IL-6 and hsCRP had little effect on the association between HMW-adiponectin and MetS (Table 2). The mechanism underlining adiponectin regulation and signaling pathway is rather complex and non-adipose factors might also be involved. Besides fat cells, adiponectin could be secreted by skeletal muscle, cardiac myocytes and endothelial cells as well [2]. Moreover, two receptors, namely AdipoR1 and AdipoR2, operate closely with AMPK or PPAR-α pathway to enhance glucose uptake and utilization in muscle, promote lipid oxidation in liver, and improve systemic insulin sensitivity [2], [31]. In addition, insulin resistance and inflammatory factors, hallmarks of MetS, are proposed to down regulate adiponectin production [2]. Previous small-scale infusion studies suggested that correlations of adiponectin with insulin sensitivity were independent of BMI [32], total adiposity measured by DEXA, visceral fat measured by magnetic resonance imaging and intramyocellular lipid measured by ^1^H-magnetic resonance spectroscopy [33]. Indeed, both enlarged size and increased number of subcutaneous adipocytes could lead to accumulation of fat in non-adipose tissue, such as skeletal muscle, liver and heart. These ectopic fats are closely related to hypoadiponectinemia and might modify its link with insulin resistance [34]. Due to the limitation of DEXA method in current study, it is not possible to exclude the effects of visceral adiposity, intramyocellular or intrahepatic lipid content or adipocytes size by adjustment of FMI, although BMI were already partitioned by fat mass and fat free mass,respectively [35]. Collectively, data from our study and others supported that HMW-adiponectin was an important biomarker for MetS in addition to many established risk factors.

### Leptin, soluble leptin receptor and metabolic syndrome

In this study, we also found strong associations between leptin and risk of MetS with adjusting BMI, inflammation and several known confounders (Table 2, Model 1 to 3). Interestingly, in contrast to the case of HMW-adiponectin, these associations could be diminished by adjusting FMI (Table 2, Model 4 and Table 3). Previously, data from a cohort study in Caucasian population suggested that leptin could predict future MetS independent of baseline BMI [14]. While leptin was associated with MetS risk in older Chinese women, but not in men after adjustment for BMI [36]. However, it was noteworthy that most existing studies utilized BMI to evaluate adiposity status, containing both fat and fat-free mass, which have different influences on metabolic disorders [28]. As an adipose-derived hormone, leptin plays a critical role in regulating energy homeostasis [13]. However, it is still not clear whether leptin resistance is one of the causes or the consequences of obesity, or the “vicious cycle” of them might be the culprit of metabolic disorders. In this study, we provide direct evidence highlighting the key role of fat mass in hyperleptinemia associated metabolic abnormalities. Our finding might be particularly important for Chinese who are characterized to have more body fat under given BMI [20], [21].

In addition to hyperleptinemia, our data revealed that reduced soluble leptin receptor (sOB-R), another component of leptin resistance, was significantly associated with an increased MetS risk independent of fat mass. sOB-R, formed by cleaving the ectodomain of membrane-anchored leptin receptors, represents the major leptin binding fraction in blood [37]. It regulates the available leptin pool and downstream signaling [38]. Few epidemiological studies have simultaneously investigated the associations of both plasma leptin and sOB-R with MetS. In a study from Framingham third generation participants (n = 362), leptin and free leptin index (molar ratio of leptin and sOB-R) were positively associated with severity of MetS after adjustment for age and sex [16]. With larger sample size, we further adjusted for diet, lifestyle, BMI/FMI, inflammatory markers and adipokines. However, our data indicated that leptin and sOB-R had unique properties and might reflect the different aspects of MetS. For instance, sOB-R was negatively associated with central obesity, elevated triglyceride and reduced HDL-C, whereas leptin was only associated with hypertriglyceridemia in multivariate regression including FMI (Table 3). Given the wide distribution in tissues containing membrane leptin receptors, it is reasonable to speculate that non-adipose mechanism(s) such as tissue specific effect might also play a role in sOB-R regulation. On the other hand, leptin was almost exclusively secreted by adipocytes, particularly subcutaneous fat. Hypertriglyceridemia might result from the limited capacity of adipose tissue to store excessive fat or up-regulated hepatic triglyceride secretion and down-regulated plasma triglyceride degradation [39], [40]. However, adjustment for HMW-adiponectin could abolish significance between sOB-R and MetS but not that between leptin and MetS (Table 2, Model 3). Indeed, findings from the Nurses' Health Study showed an inverse association between sOB-R and diabetes independent of BMI and HMW-adiponectin [15]. Kim et al. found if leptin-deficient mice have over-expressed adiponectin would result in improvements of glucose and lipid metabolism and inflammatory profile [41]. Obviously, further studies are warranted to clarify whether there are functional and/or signaling crosstalk among adiponectin, leptin and its receptor (s).

### Strengths and Limitations

This is the first study that systematically investigates the associations of HMW-adiponectin, leptin, sOB-R, body fat mass measured by DEXA, along with a wide range of inflammatory and metabolic parameters with MetS and its features in a relatively large population with both sexes. Meanwhile, we acknowledge some limitations. Because of the cross-sectional nature, no causal relationship could be established. Also, despite of the originally obesity case-control design, we adapted a cross-sectional approach in data analyses in order to enhance statistical power. Nevertheless, similar associations of HMW-adiponectin, leptin with MetS were observed in both normal and overweight/obese individuals as well as in further subgroup analyses. Obviously, our findings need to be confirmed prospectively in different populations.

In conclusion, we found strong inverse associations between HMW-adiponectin and MetS independent of adiposity, inflammatory statuses, leptin and sOB-R. Similar associations between sOB-R and MetS were also evidenced, but were weak and attenuated by HMW-adiponectin adjustment. In contrast, leptin showed strong positive associations with the risk of MetS which could be mainly explained by body fat mass. Overall, current study provides more mechanistic insights into the linkage of adipose tissue, adipokines and inflammation to metabolic syndrome and also more evidences for detection of decreased HMW-adiponectin and leptin resistance in clinical settings to prevent metabolic disorders and future diabetic and cardiovascular outcomes.

## Supporting Information

Table S1 **Characteristics of participants ^1^.** Abbreviations: MetS  =  metabolic syndrome, hsCRP  =  high sensitive C-reactive protein, HMW-adiponectin  =  high-molecular-weight adiponectin, sOB-R  =  soluble leptin receptor. ^1^ Data were Mean ± SD or Median (IQR) or Number (percentage). ^2^ Adjusted for age and sex. ^3^ Data were available for 956 participants.
(DOC)

Table S2 **Odds ratio (95% CI) of modified metabolic syndrome according to sex-specific tertile of HMW-adiponectin, leptin and sOB-R in BMI-stratified analyses ^1^.** ^1^ Definition of metabolic syndrome was modified: having 2 or more components of metabolic syndrome without central obesity. Tertiles were based on sex-specific levels in BMI-stratified subgroup. NO. of cases/control were 160/338 in normal weight group and 414/143 in overweight group. ^2^ Adjusted for the same variables in Table 2. Model 1: including BMI; Model 2: including FMI; Model 3: adjustment for log-transformed trunk fat percentage. ^3^ Data were available for 956 participants. NO. of cases/control were 147/307 in normal weight group and 369/133 in overweight group.
(DOC)

Table S3 **Odds ratio (95% CI) of metabolic syndrome according to sex-specific tertile of HMW-adiponectin, leptin and sOB-R in subgroup analyses ^1^.** ^1^ Tertiles were based on sex-specific levels in each subgroup. Adjusted for the same variables as Model 4 in Table 2, including FMI. Data were available for 956 participants. ^2^ Median concentration of FMI was 5.87 for men and 7.79 for women. Definition of metabolic syndrome was modified: having 2 or more components of metabolic syndrome without central obesity. No adjustment for FMI. ^3^ Median concentration of hsCRP was 0.87 mg/L. ^4^ Median level of HOMA-IR was 1.08.
(DOC)
