# Supplementary material for: Effects of Body Fat on the Associations of High-Molecular-Weight Adiponectin, Leptin and Soluble Leptin Receptor with Metabolic Syndrome in Chinese
Source: PLoS One. 2011 Feb 15;6(2):e16818. doi: 10.1371/journal.pone.0016818 (PMC3039650; doi:10.1371/journal.pone.0016818)
Supplement: Table S1 — Characteristics of participants 1. Abbreviations: MetS = metabolic syndrome, hsCRP = high sensitive C-reactive protein, HMW-adiponectin = high-molecular-weight adiponectin, sOB-R = soluble leptin receptor. 1 Data were Mean ± SD or Median (IQR) or Number (percentage). 2 Adjusted for age and sex. 3 Data were available for 956 participants. (DOC) [file pone.0016818.s001.doc]

**Table S1 Characteristics of participants ^1^**

| Characteristics | Non-MetS | MetS | *P* value ^2^ |
| --- | --- | --- | --- |
| N (male/female) | 612 (208/404) | 443 (197/246) |  |
| Age (yrs) | 45.4±5.5 | 46.7±5.2 | 0.0002 |
| BMI (kg/m^2^) | 22.5±3.1 | 27.6±3.3 | <.0001 |
| Newly diagnosed type 2 diabetes | 36 (5.9) | 109 (24.6) | <.0001 |
| Family history of chronic diseases | 245 (40.0) | 176 (39.7) | 0.78 |
| Current or past smoker | 146 (23.9) | 148 (33.4) | 0.27 |
| Alcohol drinker | 216 (35.3) | 167 (37.7) | 0.45 |
| Total energy intake per day | 2179 (615) | 2255 (724) | 0.06 |
| Education |  |  | 0.001 |
| 0~9 years | 147 (24.0) | 140 (31.6) |  |
| 10~12 years | 319 (52.1) | 234 (52.8) |  |
| > 12 years | 146 (23.9) | 69 (15.6) |  |
| Physical activity |  |  | 0.38 |
| Low | 59 (9.6) | 42 (9.5) |  |
| Moderate | 365 (59.6) | 245 (55.3) |  |
| High | 188 (30.7) | 156 (35.2) |  |
| Sleep |  |  | 0.71 |
| < 7 h/d | 124 (20.3) | 88 (19.9) |  |
| 7~9 h/d | 417 (68.1) | 306 (69.1) |  |
| ≥ 9 h/d | 71 (11.6) | 49 (11.1) |  |
| **Metabolic and inflammatory biomarkers** | | | |
| Waist circumference (cm) |  |  |  |
| Men | 82.5±8.2 | 96.9±8.0 | <.0001 |
| Women | 76.9±8.0 | 91.1±8.6 | <.0001 |
| Systolic blood pressure (mm Hg) | 118.0±14.2 | 134.6±17.6 | <.0001 |
| Diastolic blood pressure (mm Hg) | 74.6±9.4 | 86.4±11.2 | <.0001 |
| Fasting glucose (mmol/L) | 5.7±1.0 | 6.6±1.7 | <.0001 |
| Insulin (μU) | 8.6±4.5 | 13.4±6.4 | <.0001 |
| HOMA-IR | 0.99±0.51 | 1.58±0.74 | <.0001 |
| Triglycerides (mmol/L) | 1.06±0.56 | 2.30±1.52 | <.0001 |
| HDL cholesterol (mmol/L) |  |  |  |
| Men | 1.30±0.37 | 1.08±0.31 | <.0001 |
| Women | 1.61±0.41 | 1.28±0.32 | <.0001 |
| hsCRP (mg/L) | 0.57 (0.85) | 1.48 (1.92) | <.0001 |
| IL-6 (pg/mL) | 1.14 (0.85) | 1.65 (1.34) | <.0001 |
| **Adipokines** | | | |
| HMW-adiponectin (μg/mL) |  |  |  |
| Men | 2.47 (2.66) | 1.41 (1.59) | <.0001 |
| Women | 3.66 (3.64) | 2.28 (2.76) | <.0001 |
| Leptin (ng/mL) |  |  |  |
| Men | 2.04 (2.27) | 4.90 (3.95) | <.0001 |
| Women | 7.35 (6.76) | 14.5 (9.37) | <.0001 |
| sOB-R (ng/mL) |  |  |  |
| Men | 20.0 (6.89) | 16.7 (5.96) | <.0001 |
| Women | 19.1 (6.41) | 17.1 (5.95) | <.0001 |
| **Body composition ^3^** | | | |
| Fat mass index (kg/m^2^) |  |  |  |
| Men | 4.64±1.53 | 6.93±1.54 | <.0001 |
| Women | 7.01±1.97 | 10.0±2.18 | <.0001 |
| Trunk fat percentage (%) |  |  |  |
| Men | 10.9±3.26 | 14.9±2.35 | <.0001 |
| Women | 15.2±3.25 | 19.4±2.62 | <.0001 |

Abbreviations: MetS = metabolic syndrome, hsCRP = high sensitive C-reactive protein, HMW-adiponectin = high-molecular-weight adiponectin, sOB-R = soluble leptin receptor.

^1^ Data were Mean ± SD or Median (IQR) or Number (percentage).

^2^ Adjusted for age and sex.

^3^ Data were available for 956 participants.
