# Supplementary material for: Effects of Body Fat on the Associations of High-Molecular-Weight Adiponectin, Leptin and Soluble Leptin Receptor with Metabolic Syndrome in Chinese
Source: PLoS One. 2011 Feb 15;6(2):e16818. doi: 10.1371/journal.pone.0016818 (PMC3039650; doi:10.1371/journal.pone.0016818)
Supplement: Table S2 — Odds ratio (95% CI) of modified metabolic syndrome according to sex-specific tertile of HMW-adiponectin, leptin and sOB-R in BMI-stratified analyses 1. 1 Definition of metabolic syndrome was modified: having 2 or more components of metabolic syndrome without central obesity. Tertiles were based on sex-specific levels in BMI-stratified subgroup. NO. of cases/control were 160/338 in normal weight group and 414/143 in overweight group. 2 Adjusted for the same variables in Table 2. Model 1: including BMI; Model 2: including FMI; Model 3: adjustment for log-transformed trunk fat percentage. 3 Data were available for 956 participants. NO. of cases/control were 147/307 in normal weight group and 369/133 in overweight group. (DOC) [file pone.0016818.s002.doc]

**Table S2 Odds ratio (95% CI) of modified metabolic syndrome according to sex-specific tertile of HMW-adiponectin, leptin and sOB-R in BMI-stratified analyses ^1^**

| Adipokines | T1 | T2 | T3 | *P* for trend | *P* for interaction |
| --- | --- | --- | --- | --- | --- |
| **HMW-adiponectin** |  |  |  |  |  |
| Model 1 ^2^ |  |  |  |  |  |
| <24 kg/m^2^ | 1 | 0.83 (0.51, 1.35) | 0.39 (0.23, 0.67) | 0.0006 | 0.47 |
| ≥24 kg/m^2^ | 1 | 0.54 (0.31, 0.94) | 0.37 (0.22, 0.65) | 0.0004 |
| Model 2 ^2,3^ |  |  |  |  |  |
| <24 kg/m^2^ | 1 | 0.76 (0.46, 1.27) | 0.39 (0.22, 0.69) | 0.001 | 0.64 |
| ≥24 kg/m^2^ | 1 | 0.55 (0.31, 0.97) | 0.37 (0.21, 0.65) | 0.0005 |
| Model 3 ^2,3^ |  |  |  |  |  |
| <24 kg/m^2^ | 1 | 0.84 (0.50, 1.41) | 0.49 (0.28, 0.87) | 0.02 | 0.54 |
| ≥24 kg/m^2^ | 1 | 0.57 (0.32, 1.01) | 0.40 (0.23, 0.71) | 0.002 |
| **Leptin** |  |  |  |  |  |
| Model 1 ^2^ |  |  |  |  |  |
| <24 kg/m^2^ | 1 | 1.22 (0.73, 2.03) | 1.50 (0.88, 2.55) | 0.14 | 0.55 |
| ≥24 kg/m^2^ | 1 | 1.69 (1.02, 2.80) | 1.52 (0.89, 2.61) | 0.10 |
| Model 2 ^2,3^ |  |  |  |  |  |
| <24 kg/m^2^ | 1 | 0.89 (0.51, 1.56) | 1.10 (0.58, 2.08) | 0.75 | 0.40 |
| ≥24 kg/m^2^ | 1 | 1.53 (0.87, 2.67) | 1.27 (0.67, 2.43) | 0.42 |
| Model 3 ^2,3^ |  |  |  |  |  |
| <24 kg/m^2^ | 1 | 0.80 (0.45, 1.41) | 1.02 (0.55, 1.88) | 0.91 | 0.24 |
| ≥24 kg/m^2^ | 1 | 1.52 (0.88, 2.64) | 1.29 (0.70, 2.64) | 0.37 |
| **sOB-R** |  |  |  |  |  |
| Model 1 ^2^ |  |  |  |  |  |
| <24 kg/m^2^ | 1 | 0.64 (0.39, 1.05) | 0.58 (0.35, 0.97) | 0.03 | 0.82 |
| ≥24 kg/m^2^ | 1 | 0.84 (0.50, 1.40) | 0.66 (0.39, 1.10) | 0.11 |
| Model 2 ^2,3^ |  |  |  |  |  |
| <24 kg/m^2^ | 1 | 0.69 (0.41, 1.17) | 0.60 (0.35, 1.03) | 0.06 | 0.96 |
| ≥24 kg/m^2^ | 1 | 0.74 (0.43, 1.27) | 0.68 (0.39, 1.19) | 0.18 |
| Model 3 ^2,3^ |  |  |  |  |  |
| <24 kg/m^2^ | 1 | 0.76 (0.45, 1.29) | 0.66 (0.39, 1.15) | 0.14 | 0.96 |
| ≥24 kg/m^2^ | 1 | 0.77 (0.44, 1.32) | 0.69 (0.40, 1.20) | 0.19 |

^1^ Definition of metabolic syndrome was modified: having 2 or more components of metabolic syndrome without central obesity. Tertiles were based on sex-specific levels in BMI-stratified subgroup. NO. of cases/control were 160/338 in normal weight group and 414/143 in overweight group.

^2^ Adjusted for the same variables in Table 2. Model 1: including BMI; Model 2: including FMI; Model 3: adjustment for log-transformed trunk fat percentage.

^3^ Data were available for 956 participants. NO. of cases/control were 147/307 in normal weight group and 369/133 in overweight group.
