# Supplementary material for: Effects of Body Fat on the Associations of High-Molecular-Weight Adiponectin, Leptin and Soluble Leptin Receptor with Metabolic Syndrome in Chinese
Source: PLoS One. 2011 Feb 15;6(2):e16818. doi: 10.1371/journal.pone.0016818 (PMC3039650; doi:10.1371/journal.pone.0016818)
Supplement: Table S3 — Odds ratio (95% CI) of metabolic syndrome according to sex-specific tertile of HMW-adiponectin, leptin and sOB-R in subgroup analyses 1. 1 Tertiles were based on sex-specific levels in each subgroup. Adjusted for the same variables as Model 4 in Table 2, including FMI. Data were available for 956 participants. 2 Median concentration of FMI was 5.87 for men and 7.79 for women. Definition of metabolic syndrome was modified: having 2 or more components of metabolic syndrome without central obesity. No adjustment for FMI. 3 Median concentration of hsCRP was 0.87 mg/L. 4 Median level of HOMA-IR was 1.08. (DOC) [file pone.0016818.s003.doc]

**Table S3 Odds ratio (95% CI) of metabolic syndrome according to sex-specific tertile of HMW-adiponectin, leptin and sOB-R in subgroup analyses ^1^**

| Adipokines | case/control | T1 | T2 | T3 | *P* for trend | *P* for interaction |
| --- | --- | --- | --- | --- | --- | --- |
| **HMW-adiponectin** | | | | | | |
| Gender |  |  |  |  |  |  |
| Male | 169/179 | 1 | 0.79 (0.39, 1.60) | 0.43 (0.21, 0.91) | 0.03 | 0.47 |
| Female | 230/378 | 1 | 0.45 (0.27, 0.77) | 0.38 (0.22, 0.67) | 0.0006 |
| FMI ^2^ |  |  |  |  |  |  |
| <Median | 166/312 | 1 | 0.66 (0.41, 1.07) | 0.32 (0.19, 0.54) | <.0001 | 0.39 |
| ≥Median | 350/128 | 1 | 0.41 (0.23, 0.74) | 0.32 (0.18, 0.58) | 0.0002 |
| hsCRP ^3^ |  |  |  |  |  |  |
| <Median | 112/368 | 1 | 0.69 (0.36, 1.34) | 0.61 (0.30, 1.27) | 0.17 | 0.68 |
| ≥Median | 287/189 | 1 | 0.52 (0.29, 0.91) | 0.36 (0.20, 0.65) | 0.0007 |
| HOMA-IR ^4^ |  |  |  |  |  |  |
| <Median | 112/366 | 1 | 0.85 (0.46, 1.57) | 0.34 (0.16, 0.72) | 0.007 | 0.23 |
| ≥Median | 287/191 | 1 | 0.48 (0.27, 0.87) | 0.48 (0.26, 0.87) | 0.02 |
| **Leptin** |  |  |  |  |  |  |
| Gender |  |  |  |  |  |  |
| Male | 169/179 | 1 | 1.84 (0.81, 4.22) | 2.17 (0.77, 6.08) | 0.16 | 0.72 |
| Female | 230/378 | 1 | 1.07 (0.56, 2.01) | 1.48 (0.70, 3.15) | 0.26 |
| FMI ^2^ |  |  |  |  |  |  |
| <Median | 166/312 | 1 | 1.70 (1.02, 2.82) | 2.27 (1.37, 3.77) | 0.002 | 0.62 |
| ≥Median | 350/128 | 1 | 2.16 (1.26, 3.68) | 2.00 (1.17, 3.42) | 0.009 |
| hsCRP ^3^ |  |  |  |  |  |  |
| <Median | 112/368 | 1 | 0.70 (0.29, 1.71) | 1.79 (0.70, 4.86) | 0.09 | 0.11 |
| ≥Median | 287/189 | 1 | 0.98 (0.54, 1.76) | 1.01 (0.49, 2.10) | 0.98 |
| HOMA-IR ^4^ |  |  |  |  |  |  |
| <Median | 112/366 | 1 | 0.99 (0.41, 2.40) | 1.35 (0.50, 3.63) | 0.46 | 0.52 |
| ≥Median | 287/191 | 1 | 0.75 (0.40, 1.38) | 0.60 (0.28, 1.29) | 0.19 |
| **sOB-R** | | | | | | |
| Gender |  |  |  |  |  |  |
| Male | 169/179 | 1 | 0.46 (0.22, 0.95) | 0.64 (0.30, 1.36) | 0.23 | 0.65 |
| Female | 230/378 | 1 | 0.70 (0.41, 1.19) | 0.82 (0.47, 1.42) | 0.43 |
| FMI ^2^ |  |  |  |  |  |  |
| <Median | 166/312 | 1 | 0.54 (0.33, 0.89) | 0.45 (0.27, 0.74) | 0.002 | 0.36 |
| ≥Median | 350/128 | 1 | 0.93 (0.54, 1.60) | 0.75 (0.43, 1.28) | 0.29 |
| hsCRP ^3^ |  |  |  |  |  |  |
| <Median | 112/368 | 1 | 0.59 (0.30, 1.16) | 0.80 (0.39, 1.64) | 0.43 | 0.87 |
| ≥Median | 287/189 | 1 | 0.63 (0.36, 1.12) | 0.57 (0.32, 1.03) | 0.07 |
| HOMA-IR ^4^ |  |  |  |  |  |  |
| <Median | 112/366 | 1 | 0.40 (0.20, 0.79) | 0.90 (0.46, 1.77) | 0.61 | 0.13 |
| ≥Median | 287/191 | 1 | 0.71 (0.40, 1.27) | 0.63 (0.35, 1.14) | 0.13 |

^1^ Tertiles were based on sex-specific levels in each subgroup. Adjusted for the same variables as Model 4 in Table 2, including FMI. Data were available for 956 participants.

^2^ Median concentration of FMI was 5.87 for men and 7.79 for women. Definition of metabolic syndrome was modified: having 2 or more components of metabolic syndrome without central obesity. No adjustment for FMI.

^3^ Median concentration of hsCRP was 0.87 mg/L.

^4^ Median level of HOMA-IR was 1.08.
